# 

**Published:** 1892-05-07

**Authors:** 


					? PRlCrTwOPENCE^ o
^93, Vol. XII.?May 7th, 1892.
at tie General Post Office as a Newspaper lor
^"OUHBion in the United Kingdom and Abroad. 1
*
WITH EXTRA NURSING SUPPLEMENT.
Per Annum, 8s. 6cL, post free.
Monthly Farts, 6d.; post free, 8d.
Ditto per Annum, 8s., post free.
No. 293. Vol. XII.] Saturday,
May 7th, 1892.
[Twopence.

				

